# Operative technique: sutureless type II hybrid arch repair for acute type A aortic dissection

**DOI:** 10.1093/icvts/ivaf081

**Published:** 2025-04-08

**Authors:** John Chien-Hwa Chang, Shih-Ming Huang, Ing-Heng Hii, Chi-Fu Cheng, Pei-Chei Lu, Yi-Tso Cheng

**Affiliations:** Division of Cardiovascular Surgery, Dalin Tzu Chi Hospital, Buddhist Tzu Chi Medical Foundation, Dalin, Taiwan; Department of Medicine, Tzu Chi University, Hualien, Taiwan; Division of Cardiovascular Surgery, Dalin Tzu Chi Hospital, Buddhist Tzu Chi Medical Foundation, Dalin, Taiwan; Department of Medicine, Tzu Chi University, Hualien, Taiwan; Division of Cardiovascular Surgery, Dalin Tzu Chi Hospital, Buddhist Tzu Chi Medical Foundation, Dalin, Taiwan; Division of Cardiovascular Surgery, Dalin Tzu Chi Hospital, Buddhist Tzu Chi Medical Foundation, Dalin, Taiwan; Division of Cardiovascular Surgery, Dalin Tzu Chi Hospital, Buddhist Tzu Chi Medical Foundation, Dalin, Taiwan; Department of Medicine, Tzu Chi University, Hualien, Taiwan; Division of Cardiac Surgery, Taichung Tzu Chi Hospital, Buddhist Tzu Chi Medical Foundation, Taichung, Taiwan

**Keywords:** sutureless aortic anastomosis, acute type A aortic dissection, hybrid arch repair

## Abstract

**OBJECTIVES:**

Hybrid arch repair improved surgical outcomes in aneurysmal disease. Sutureless anastomosis using an intraluminal ringed graft and stent graft bridging has been reported. We incorporate the vascular ring connector, angiography-assisted sutureless telescoping anastomosis technique, and thoracic endovascular aortic repair, rendering the hybrid arch repair for acute type A aortic dissection sutureless. Herein, we presented our sutureless procedure for acute type A aortic dissection.

**METHODS:**

Between January 2022 and April 2023, 19 patients who underwent sutureless type II hybrid arch repair were enrolled. The surgical procedures were described. The preoperative demographics, operative details, postoperative outcomes and follow-up results were retrospectively collected.

**RESULTS:**

Nineteen patients with a median age of 62 (interquartile range [IQR]: 10.5) and male dominant in 73.7% were recorded. The sutureless type II hybrid arch repair was performed in a median operative time of 397 min (IQR: 111.5), with a cardiopulmonary bypass time of 184 min (IQR: 52.5). The fully sutureless type II hybrid arch repair further reduced the abovementioned times. In-hospital death was two in 10.5%. Seventeen discharged patients had regular follow-ups in a median of 553 days (IQR: 129). The serial computed tomography scan revealed all reconstructed arch vessels were patent, and positive aortic remodelling was observed at the arch and thoracic endoprosthesis levels at 100% and 94.2%, respectively.

**CONCLUSIONS:**

Sutureless type II hybrid arch repair is feasible, demonstrating complete procedural success and favourable postoperative outcomes in mid-term follow-up. Long-term monitoring is necessary to assess this procedure’s durability and potential complications.

## GRAPHICAL ABSTRACT

**Figure:**
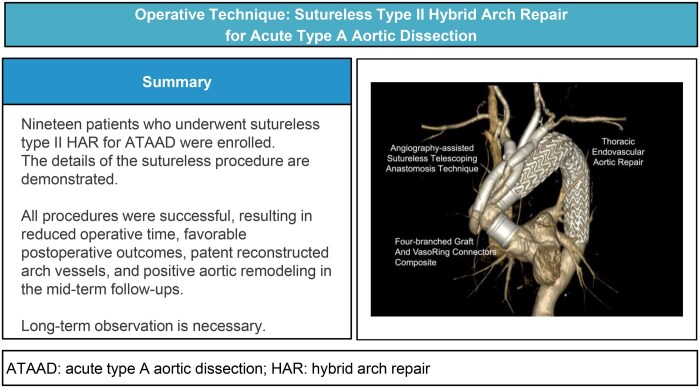

## INTRODUCTION

Hybrid arch repair (HAR) combines open surgery to create a landing zone for endoprostheses to treat arch aneurysms, proving safe and effective in high-risk patients [1]. A type II HAR is used when there is no aortic zone 0 landing, requiring reconstruction of the ascending aorta.

The vascular ring connector (VRC [Sunwei Technology Co, Taipei, Taiwan]) is reported for its sutureless design in graft anastomosis of dissected vessels, significantly reducing both anastomosis time and the risk of bleeding [2–5]. It is commercially available and reimbursed by Taiwan’s National Health Insurance since 2009. Stent graft-assisted anastomosis using the sutureless telescoping anastomosis technique (STAT) has been reported for arch vessel reconstruction in aneurysmal disease [6–8]. We modified this technique by incorporating intraoperative angiography to manage aortic dissection pathology.

We developed a sutureless approach to treat acute type A aortic dissection (ATAAD) using type II HAR and the techniques above. This approach involves ascending aortic replacement with a four-branch graft facilitated by the VRC in the aortic anastomosis, arch vessel reconstruction using angiography-assisted STAT, and thoracic endovascular aortic repair (TEVAR) to address the remaining aortic arch and descending aorta—making the entire procedure sutureless. This study aims to present the outcomes of this sutureless type II HAR for ATAAD.

## MATERIALS AND METHODS

Between January 2022 and April 2023, 20 patients with ATAAD underwent total arch procedures in Dalin Tzu Chi Hospital. Among them, 19 patients (95%) who underwent sutureless type II HAR were enrolled, including nine who underwent a fully sutureless HAR. The clinical variables were retrospectively reviewed, including preoperative patient demographics, operative details, postoperative outcomes and discharge follow-up results. All patients were followed up until July 2024. The Institutional Review Board of Dalin Tzu Chi Hospital approves establishing and monitoring the ongoing use of such databases and biobanks. The stored data and biobanks are consistent with the requirements outlined in the World Medical Association Declaration of Taipei. The Study number is B11302002A. Informed consent was waived for a retrospective study with a 6-month window period.

### Surgical procedures

Patients were intubated and placed in a supine position. A full sternotomy was made. The innominate vein was divided. Cardiopulmonary bypass was initiated with two arterial cannulations via the right axillary or innominate artery and femoral artery and one venous drainage at the right atrium. Moderate systemic hypothermia at 30°C is utilized. We divided the sutureless type II HAR into three stages.

### First stage: ascending aorta replacement using the VRC graft composite

**Figure 1:**
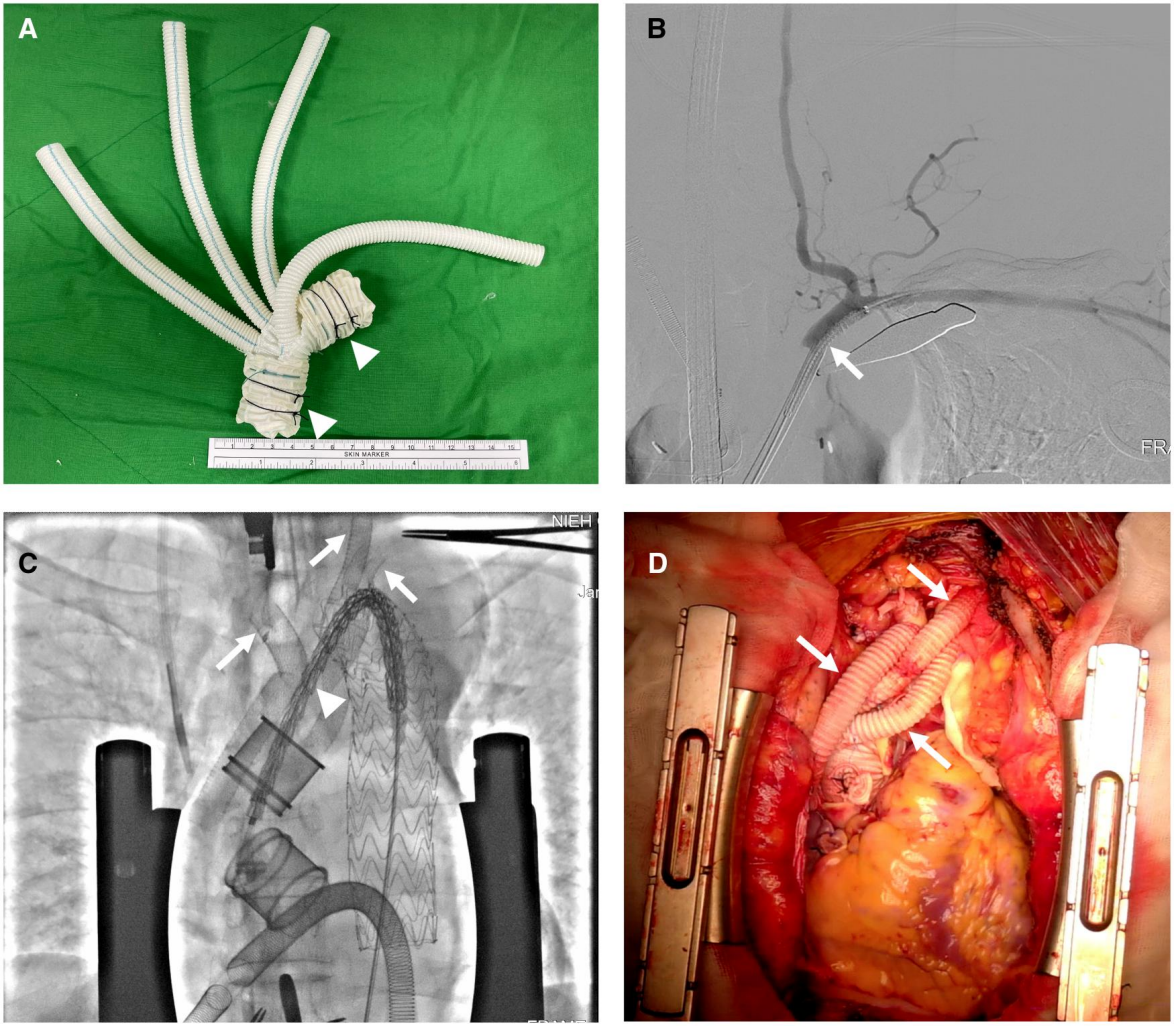
Intraoperative images. (**A**) The composite of the four-branch graft and vascular ring connectors was constructed using a cuff-fold modification, where the graft is passed through the vascular ring connector and then folded back at both ends (arrowhead). (**B**) Left subclavian angiography confirmed the true lumen and the origin of the left vertebral artery, with the stent graft (white arrow) for deployment. (**C**) Completion of the arch vessel reconstruction using angiography-assisted sutureless telescoping anastomosis technique (white arrows) and proximal endoprosthesis (arrowhead) ready for deployment under fluoroscopy. (**D**) Final surgical result

**Figure 2:**
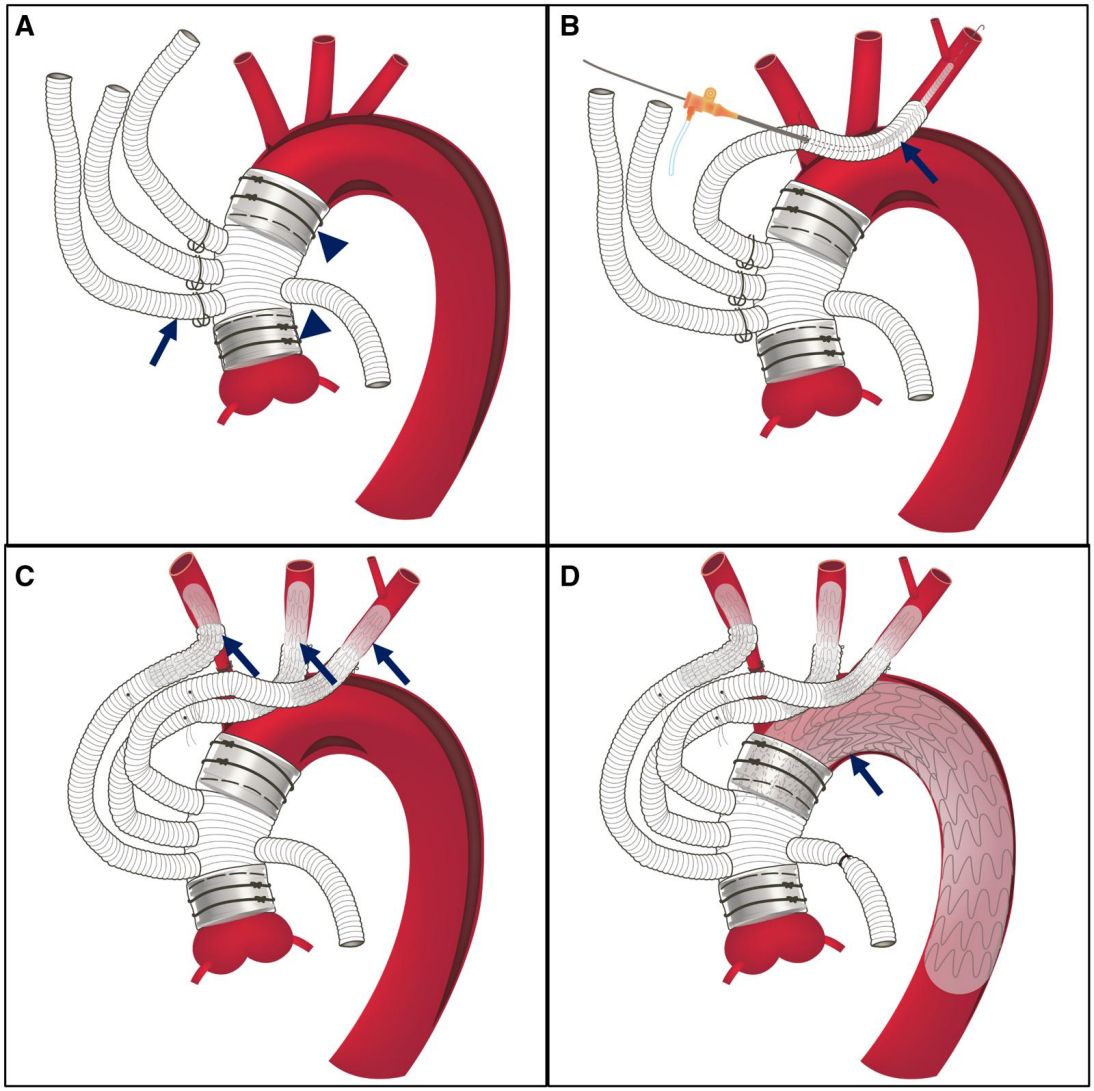
Schematic presentation of the sutureless type II hybrid arch repair, which involves replacing the ascending aorta using a four-branch graft (black arrows) and vascular ring connectors (arrowheads) composite (**A**). Arch vessel reconstruction using an introduced sheath with a stent graft (black arrow) prepared for deployment (**B**). Completing the arch vessel reconstruction using the sutureless telescoping anastomosis technique (black arrows) (**C**). Remaining arch and descending aorta reconstruction using thoracic endovascular aortic repair (black arrow) (**D**)

Following the aortic cross-clamp, a partial transverse aortotomy was made at the mid-ascending aorta. The inner diameter of the proximal aorta was measured for the size of the four-branched graft (J-graft, Japan Lifeline, Tokyo) and VRCs. The same size of the four-branched graft and VRCs were chosen. A cuff-fold modification that passes the graft through the VRC and then folds it back as a cuff fold is suggested to create the VRC graft composite (Fig. 1A). The VRC graft composite facilitated the aortic anastomosis by introducing it into the aorta and securing both together using surgical tapes (Fig. 2A). Selective antegrade cerebral perfusion was applied during distal aortic anastomosis. After replacing the ascending aorta, systemic and cardiac perfusion was resumed by redirecting the cardiopulmonary flow from femoral cannulation to the perfusion branch of the four-branch graft. Systemic temperature was rewarmed.

### Second stage: angiography-assisted STAT reconstructs the arch vessel under continuous perfusion

The detailed procedure for STAT for arch vessel reconstruction in aneurysmal disease has been described [8]. In dissection cases, we use intraoperative angiography to confirm the true lumen engagement (Fig. 1B). An 11-French sheath was inserted into a side branch of the four-branched graft with a preplaced purse-string suture. The sheath was advanced into the target vessel under fluoroscopy using the Seldinger technique. Echo-guided puncture is required if the target vessel is dissected. Digital subtraction angiography (DSA) confirmed the true lumen and the origin of primary branches, such as the left vertebral artery. A suitable-sized stent graft was introduced and deployed to connect the side branch and the target vessel (Fig. 2B). Post-dilatation using an 8-mm or larger balloon was performed. Two stitches were applied to secure the connection, incorporating the side branch, stent graft, and the target vessel. The preplaced purse-string suture was used for de-airing and closure. After the procedure, perfusion to the target vessel was immediately resumed. The arch vessels were individually reconstructed in the order of the left subclavian artery (LSA), the left common carotid artery (LCCA) and the innominate artery (IA) (Fig. 2C).

The Viabahn stent graft (W.L. Gore & Associates, Inc. Newark, Delaware) was used in our series to perform the STAT. The side branch diameters of the four-branched graft (J-graft, Japan Lifeline, Tokyo) are 11-9-9-9 mm. A 1- to 4-mm oversize is suitable for the side branch, while a 1- to 2-mm oversize is acceptable for the target vessel. In our series, an 11-mm stent graft was most commonly used for LSA reconstruction, while 10-mm and 13-mm stent grafts were used for LCCA and IA reconstruction, respectively (Table 1). If the target vessel is <8-mm, an 8-mm stent graft can be preemptively deployed inside as protection, followed by a 10-mm stent graft to complete the procedure. Aneurysmal changes are commonly encountered during IA reconstruction. Surgical anastomosis can be performed, or angiography-assisted STAT can be done using an iliac limb stent graft. In our series, a 16-mm Endurant iliac limb stent graft (Medtronic, Minneapolis, Minnesota) has been used for IA reconstruction in one patient.

**Table 1:** Detailed size and distribution of surgical supplies

	No.	Size, mm (IQR)
Four-branch graft	19 (100%)	28 (4)
Vascular ring connector		
Proximal aortic anastomosis	15 (78.9%)	28 (3)
Distal aortic anastomosis	17 (89.5%)	28 (3)
Arch vessel reconstruction	58 (100%)	
By angiography-assisted STAT	45 (77.6%)	
Deployed stent grafts by arch vessel (*n*)		
Innominate artery (19)	11 (57.9%)	13 (0)
Left common carotid artery (19)	18 (94.7%)	10 (0)
Left subclavian artery (19)	16 (84.2%)	11 (2)
Thoracic endovascular aortic repair	19 (100%)	28 (3.5)

STAT: sutureless telescoping anastomosis technique.

### Third stage: TEVAR for arch and descending aorta reconstruction

The thoracic endoprostheses, introduced via femoral cannulation, were deployed to bridge the four-branched graft and the descending aorta under fluoroscopy (Fig. 2D). The thoracic endoprosthesis’s size matched the descending aorta at the aortic zone 4 [9] without oversizing (Fig. 1C). The used thoracic endoprosthesis included Gore TAG (W.L. Gore & Associates, Inc. Newark, Delaware) in 11 patients and Valiant endoprosthesis (Medtronic, Minneapolis, Minnesota) in eight patients.

After TEVAR, the femoral artery was repaired. Systemic and cardiac perfusion and the rewarming process resumed at the beginning of the second stage, and weaning off cardiopulmonary bypass was done after the third stage. A sutureless type II HAR can be performed (Figs 1D and 2D). The size and distribution of the four-branch grafts, Viabanh stent grafts and distal thoracic endoprostheses are listed in Table 1.

Fully sutureless type II HAR was achieved in nine patients (47.4%). A surgical video overview of a fully sutureless type II HAR is provided (Supplementary Video S1).

### Statistical analysis

The categorical variables were presented with frequencies and percentages, while the continuous variables used the median with the interquartile range (IQR). All data were analysed using MedCalc Statistical Software version 22.021.

## RESULTS

### Patient demographics

Nineteen patients enrolled with a median age of 62 (IQR: 10.5), with males predominant at 14 (73.7%). The baseline characteristics and preoperative conditions are summarized in Table 2. All patients presented Stanford type A aortic dissection. Among them, 17 were classified as DeBakey type I (89.5%), while two had DeBakey type II with arch aneurysm. Five patients (26.3%) presented with preoperative cerebral ischaemia. The indication for type II HAR is arch entry tear in 11 (57.9%), ascending aorta entry tear with arch vessel dissection or arch aneurysm in 4 (21.1%) and 2 (10.5%), respectively, and retrograde dissection from the descending aorta in 2 (10.5%).

**Table 2:** Patient demographics and characteristics

Characteristics	All
Number of patients	19
Age, years (IQR)	62 (10.5)
Male sex	14 (73.7%)
Body mass index (IQR)	23.7 (4.55)
Hypertension	14 (73.7%)
Diabetes	2 (10.5%)
Dyslipidaemia	1 (5.3%)
Emergency	17 (89.5%)
Aortic dissection classification	
Stanford type A	19 (100%)
DeBakey type I	17 (89.5%)
DeBakey type II with arch aneurysm	2 (10.5%)
Preoperative morbidity	
Shock status	7 (36.8%)
Hemopericardium	10 (52.6%)
Cerebral ischaemia	5 (26.3%)
Mesenteric ischaemia	1 (5.3%)
Limb ischaemia	1 (5.3%)
Indication for type II hybrid arch repair	
Arch entry tear	11 (57.9%)
Ascending aorta entry tear with Arch vessel dissection	4 (21.1%)
Ascending aorta entry tear with Arch aneurysm	2 (10.5%)
Descending aorta entry tear	2 (10.5%)

IQR: interquartile range.

### Operative details

All procedures for attempting sutureless type II HAR were successful. The median operative time, cardiopulmonary bypass time and aortic cross-clamp time were 397 min (IQR: 111.5), 184 min (IQR: 52.5) and 68 min (IQR: 66.5), respectively. The fully sutureless type II HAR can further reduce the abovementioned times (Table 3).

**Table 3:** Operative details

Characteristics	All	Fully sutureless type II HAR
Number of patients	19	9
Operative variables		
Operative time, min (IQR)	397 (111.5)	344 (134.75)
CPB time, min (IQR)	184 (52.5)	180 (44.25)
Cross-clamp time, min (IQR)	68 (66.5)	44 (8.25)
SACP time, min (IQR)	10 (12.87)	4.08 (6)

CPB: cardiopulmonary bypass; HAR: hybrid arch repair; IQR: interquartile range; SACP: selective antegrade cerebral perfusion.

Table 1 presents the details of the arch vessel reconstruction. Owing to one patient having an aberrant right subclavian artery requiring individual surgical anastomosis, 19 patients underwent reconstruction of a total of 58 arch vessels. Of these, 45 vessels (77.6%) underwent angiography-assisted STAT. The LCCA and LSA had a high proportion of angiography-assisted STAT in 94.7% and 84.2%, respectively.

### Postoperative outcomes

The postoperative outcomes are listed in Table 4. Seventeen patients (89.5%) survived to discharge, while two in-hospital deaths (10.5%) were due to mesenteric ischaemia. In discharged patients, the median ventilation time and hospital stay were 27.2 h (IQR: 8.35) and 26 days (IQR: 31). Preoperative cerebral ischaemia had a strong impact, with four out of six patients on ventilation exceeding 96 h, and four out of seven patients on hospital stays surpassing 30 days. One patient (5.3%) with preoperative abdominal symptoms received intraoperative superior mesenteric artery (SMA) stenting and survived to discharge. No resternotomy for bleeding, no new-onset postoperative cerebral ischaemia, and no paraplegia were noted in our series.

**Table 4:** Postoperative outcomes

Postoperative outcomes	
In-hospital mortality	2 (10.5%)
Discharged patients	17 (89.5%)
Ventilation time, h (IQR)	27.2 (188.2)
Ventilation >96 h	6 (35.3%)
Hospital stay, days (IQR)	26 (31)
Hospital stay >30 days	7 (36.8%)
Major morbidities	
Mesenteric ischaemia	3
Preoperative cerebral ischaemia	5
Pneumonia	1
Deep sternal infection	1
Re-sternotomy for bleeding	0
Postoperative cerebral ischaemia	0
Paraplegia	0

IQR: interquartile range.

### Follow-up results

All discharged patients had complete follow-ups until July 2024, including serial computed tomography (CT) scans in postoperative 1, 6 and 12 months. The median follow-up time is 553 days (IQR: 129), with one late mortality for pneumonia in a disabled patient due to preoperative stroke. Serial follow-up CT scans evaluated 52 reconstructed arch vessels, all remaining patent without new dissections (Table 5). Preoperative CT scans identified 18 dissected arch vessels, which were reconstructed using surgical anastomosis in six cases (33.3%) and angiography-assisted STAT in 12 (66.6%). Four residual vessel dissections were observed, with three in the surgical anastomosis group (50%) and one in the angiography-assisted STAT group (8.3%).

**Table 5:** Outcomes of the reconstructed arch vessel in discharged patients

	All	Surgical anastomosis	Angiography-assisted STAT
Number of reconstructed arch vessels	52	11	41
Patency	100%	100%	100%
Reconstructed arch vessel			
Innominate artery	17	7 (41.2%)	10 (58.8%)
Left common carotid artery	17	1 (5.9%)	16 (94.1%)
Left subclavian artery	17	2 (11.8%)	15 (88.2%)
Aberrant right subclavian artery	1	1	0
Outcomes			
Pre-operative dissection	18 (34.6%)	6 (54.5%)	12 (29.3%)
Residual dissection	4 (22.2%)	3 (50%)	1 (8.3%)
New dissection	0	0	0
Reintervention	2 (3.8%)	1 (9.1%)	1 (2.4%)

STAT: sutureless telescoping anastomosis technique.

Serial follow-up CT scans revealed positive aortic remodelling with the thrombosed false lumen at the aortic arch and TEVAR level (zone 4) in 100% and 94.1% of cases, with a worsening trend towards the aortic zones 5 and 6 by 47.1% and 29.4%, respectively. All discharged patients are prescribed antiplatelet medication unless contraindicated. No new-onset neurological deficits were detected during follow-up.

## DISCUSSION

The development of endoprosthesis has made hybrid surgeries combining conventional surgery and stent procedures feasible [1, 10]. The type II HAR was proposed to address arch aneurysms extending towards the aortic zone 0 [1]. With true lumen verified via angiography, we extended the type II HAR to ATAAD. Utilizing the VRC, angiography-assisted STAT, and TEVAR allows the procedure to be sutureless and arch reconstruction under continuous perfusion during the rewarming process. Our series demonstrated the feasibility of the procedure, highlighted by reduced operative time, and favourable follow-up outcomes, including full patency of all reconstructed vessels and positive aortic remodelling. Since introducing sutureless type II HAR in 2022, we have utilized this approach for all patients with ATAAD requiring arch management.

The details of the VRC were reported by Wei *et al.* [3] in 2009. Surgical tapes that tied around the overlapping region of the aorta and VRC graft composite provided a sutureless and blood-sealed anastomosis. Concerns were raised about potential pressure necrosis on the aortic wall due to surgical tapes. However, no pseudoaneurysms were reported in mid-term follow-ups [2, 4, 5], nor was necrotic aortic wall observed from our patients who underwent re-do operations for arch dissection aneurysms after prior ascending aortic replacement using the VRC. Dislodgement of the intraluminal ringed graft is another issue [11] managed with the VRC’s furrow design [3]. We use a cuff-fold modification, ensuring all the interior surfaces are grafted. With this modification, one layer of the graft and aorta remains outside the VRC, as originally designed. Although the VRC has been reported for use in a patient with Marfan’s syndrome, its application in young patients with hereditary thoracic aortic disease should be cautiously approached. In our experience, the mid-term outcome of VRCs to facilitate aortic anastomosis in dissected vessels is favourable. More follow-ups are required to demonstrate its safety and long-term durability.

The severity of the dissected aorta and arch vessels limited the applicability of fully sutureless HAR. Surgical anastomosis was required in cases where the dissection inlet was low or aortic root replacement was necessary. Additionally, when an aneurysmal IA was encountered without a suitable stent graft or when an extra-anatomical graft bypass was needed for arch vessel reconstruction, surgical anastomosis can be done. Type II HAR can be performed by combining surgical anastomosis with sutureless techniques, achieving comparable outcomes in our series.

Routine stent bridging to the arch vessels in open anastomosis was reported as a safe technique, but over-stenting is a concern [12]. The branch-stented anastomosis of *in-situ* fenestrated thoracic endoprosthesis has shown comparable safety [13, 14]; however, structure integrity and possible type 3 endoleak remain the issues [15]. We employed angiography-assisted STAT to prevent over-stenting and structural damage to the thoracic endoprosthesis, achieving 100% vessel patency in 1-year observation. Ongoing follow-up is necessary.

Angiography-assisted STAT is an end-to-side anastomosis with several advantages. First, it can be performed under continuous perfusion, eliminating the need for deep hypothermic cardiac arrest or selective antegrade or retrograde cerebral perfusion strategies. Second, STAT minimizes brain perfusion interruption during the stent deployment and resumes perfusion immediately after de-airing, preventing cerebral ischaemia during reconstruction. The intraoperative DSA ensures the true lumen, confirms the end of the dissected false lumen and stents the vessel accordingly. Arch vessel reconstruction under angiography resulted in a few residual arch vessel dissections by 8.4% and no new dissections. Reconstructing the arch vessels during the rewarming process accelerates the operation.

Positive aortic remodelling with the thrombosed false lumen showed benefits in decreased late reoperation for the residual dissected aorta [16, 17]. By using sutureless type II HAR, positive aortic remodelling was observed in 100% of cases at the aortic arch and 94.1% at the thoracic endoprosthesis level. Distal stent graft-induced new entries after the TEVAR is a problem [18]. We avoid that by not oversizing the thoracic endoprosthesis, deploying it under fluoroscopy, and aiming to land at a straight descending aorta for less spring-back force [19]. Our series found no distal new entries in limited follow-up; regular monitoring is required [20]. Limited TEVAR at aortic zone 4 decreased the possibility of spinal ischaemia [21]. No paraplegia was noted in our series.

Organ ischaemia remains the primary issue of morbidity and mortality following surgery for ATAAD [22, 23]. In our series, preoperative cerebral ischaemia had a strong impact on postoperative ventilator time and hospital stay. Mesenteric ischaemia is difficult [24]. Two patients in our series died from it. Hybrid operating concepts had benefits in intraoperative intervention [25]. After that, we incorporated intraoperative DSA for patients with symptoms or dissected SMA after completing sutureless type II HAR. One patient, case 17, who presented preoperative abdominal pain, had SMA malperfusion on intraoperative DSA and had an SMA stenting. He recovered well. More work had to be followed to address the safety and efficacy of intraoperative intervention.

This single-centre retrospective study for a new procedure may have several limitations, including potential biases in patient selection, lack of external validation and concerns regarding reproducibility and durability. The limited availability of the VRC may restrict its use. Limited follow-up is a major problem in developing a new procedure. Long-term follow-up is required to address this sutureless procedure’s durability and potential complications. Nevertheless, sutureless type II HAR demonstrated favourable operative details, postoperative outcomes and mid-term follow-ups in discharged patients.

## CONCLUSIONS

In our series with limited follow-up, the sutureless type II HAR facilitates the arch procedure for ATAAD in reduced operative times without compromising postoperative outcomes. Patent arch vessels with few residual dissections and favourable aortic remodelling in the follow-up present a positive impact. Long-term observation is needed to address its durability.

## Supplementary Material

ivaf081_Supplementary_Data

## Data Availability

All data are incorporated into the article and its online supplementary material.

## ABBREVIATIONS

ATAAD: Acute type A aortic dissection
CT: Computed tomography
DSA: Digital subtraction angiography
HAR: Hybrid arch repair
IA: Innominate artery
IQR: Interquartile range
LCCA: Left common carotid artery
LSA: Left subclavian artery
SMA: Superior mesenteric artery
STAT: Sutureless telescoping anastomosis technique
TEVAR: Thoracic endovascular aortic repair
VRC: Vascular ring connector
